# Supplementary material for: Modeling glioblastoma heterogeneity as a dynamic network of cell states
Source: Mol Syst Biol. 2021 Sep 16;17(9):e10105. doi: 10.15252/msb.202010105 (PMC8444284; doi:10.15252/msb.202010105)
Supplement: Supplementary file 6 — Source Data for Figure 5 [file MSB-17-e10105-s004.zip › Figure5A_sourcedata/GSEA_3017/hallmarks_stateF.GseaPreranked.1621934628066/HALLMARK_COMPLEMENT.html]

Details for gene set HALLMARK\_COMPLEMENT[GSEA]

|  || Dataset | state33017 |
| Phenotype | NoPhenotypeAvailable |
| Upregulated in class | na\_pos |
| GeneSet | HALLMARK\_COMPLEMENT |
| Enrichment Score (ES) | 0.4732535 |
| Normalized Enrichment Score (NES) | 2.259378 |
| Nominal p-value | 0.0 |
| FDR q-value | 0.0010107692 |
| FWER p-Value | 0.004 |
Table: GSEA Results Summary

  

Fig 1: Enrichment plot: HALLMARK\_COMPLEMENT      
 Profile of the Running ES Score & Positions of GeneSet Members on the Rank Ordered List

  

| PROBE | GENE SYMBOL | GENE\_TITLE | RANK IN GENE LIST | RANK METRIC SCORE | RUNNING ES | CORE ENRICHMENT || 1 | MT3 |  |  | 1 | 2.229 | 0.1121 | Yes |
| 2 | CLU |  |  | 5 | 1.852 | 0.2043 | Yes |
| 3 | LGALS3 |  |  | 16 | 1.261 | 0.2634 | Yes |
| 4 | F3 |  |  | 41 | 0.978 | 0.3020 | Yes |
| 5 | LRP1 |  |  | 92 | 0.765 | 0.3181 | Yes |
| 6 | SCG3 |  |  | 130 | 0.698 | 0.3367 | Yes |
| 7 | CEBPB |  |  | 190 | 0.601 | 0.3405 | Yes |
| 8 | TIMP2 |  |  | 196 | 0.590 | 0.3680 | Yes |
| 9 | CA2 |  |  | 243 | 0.558 | 0.3755 | Yes |
| 10 | C1R |  |  | 249 | 0.554 | 0.4012 | Yes |
| 11 | S100A13 |  |  | 261 | 0.544 | 0.4237 | Yes |
| 12 | LAMP2 |  |  | 308 | 0.506 | 0.4286 | Yes |
| 13 | LIPA |  |  | 364 | 0.483 | 0.4282 | Yes |
| 14 | HSPA5 |  |  | 373 | 0.480 | 0.4488 | Yes |
| 15 | CTSL |  |  | 376 | 0.479 | 0.4721 | Yes |
| 16 | PRCP |  |  | 426 | 0.460 | 0.4733 | Yes |
| 17 | CTSO |  |  | 584 | 0.403 | 0.4229 | No |
| 18 | DGKG |  |  | 650 | 0.385 | 0.4131 | No |
| 19 | DOCK4 |  |  | 668 | 0.380 | 0.4246 | No |
| 20 | ADAM9 |  |  | 722 | 0.365 | 0.4192 | No |
| 21 | CD59 |  |  | 897 | 0.326 | 0.3573 | No |
| 22 | C1S |  |  | 1087 | 0.294 | 0.2871 | No |
| 23 | LGMN |  |  | 1110 | 0.291 | 0.2919 | No |
| 24 | CPM |  |  | 1160 | 0.284 | 0.2841 | No |
| 25 | FN1 |  |  | 1238 | 0.275 | 0.2634 | No |
| 26 | CTSD |  |  | 1245 | 0.275 | 0.2745 | No |
| 27 | USP8 |  |  | 1253 | 0.274 | 0.2852 | No |
| 28 | PSMB9 |  |  | 1347 | 0.262 | 0.2566 | No |
| 29 | PFN1 |  |  | 1582 | -0.282 | 0.1654 | No |
| 30 | USP14 |  |  | 1607 | -0.287 | 0.1691 | No |
| 31 | CALM3 |  |  | 2022 | -0.516 | 0.0087 | No |
| 32 | SERPINE1 |  |  | 2161 | -0.843 | -0.0108 | No |
| 33 | TFPI2 |  |  | 2209 | -1.026 | 0.0198 | No |
Table: GSEA details [plain text format]

  

Fig 2: HALLMARK\_COMPLEMENT: Random ES distribution      
 Gene set null distribution of ES for **HALLMARK\_COMPLEMENT**
